# The effect of dietary level of two inulin types differing in chain length on biogenic amine concentration, oxidant-antioxidant balance and DNA repair in the colon of piglets

**DOI:** 10.1371/journal.pone.0202799

**Published:** 2018-09-07

**Authors:** Marcin Barszcz, Marcin Taciak, Anna Tuśnio, Ewa Święch, Ilona Bachanek, Paweł Kowalczyk, Andrzej Borkowski, Jacek Skomiał

**Affiliations:** 1 Department of Animal Nutrition, The Kielanowski Institute of Animal Physiology and Nutrition, Polish Academy of Sciences, Jabłonna, Poland; 2 Geomicrobiology Laboratory, Faculty of Geology, University of Warsaw, Warsaw, Poland; University of Illinois, UNITED STATES

## Abstract

The effect of dietary level of two types of inulin on amine concentration, redox status and DNA glycosylase activity in the colon of piglets was investigated. Seven groups of piglets were fed diets without inulin addition (control) or with 1%, 2% or 3% inulin with an average degree of polymerisation of 10 (IN10) or 23 (IN23) for 40 days. The 2% and 3% IN10 diets increased tryptamine concentration in the proximal colon, while methylamine concentration in the distal colon was increased by the 1% and 3% IN10 diets. The 1% and 2% IN23 diets increased phenylethylamine and methylamine concentration in the proximal colon, respectively, while 1,7-diaminoheptane content was increased by both diets. Its concentration in the middle and distal colon was increased by the 1% and 2% IN23 diet, respectively. There was no improvement in the oxidant-antioxidant balance in colonic digesta of piglets fed IN10 and IN23 diets. Piglets fed IN10 diets had lower 1,*N*^6^-etheno-2’-deoxyadenosine excision activity in each colon segment, as compared with the control group. It was also reduced by the 2% and 3% IN23 diets in the proximal colon, while in the middle and distal colon by all IN23 diets. Feeding all IN10 and IN23 diets reduced 3,*N*^4^-etheno-2’-deoxycytidine and 8-oxo-deoxyguanosine excision activities in each colon segment. Feeding IN10 and IN23 diets neither decreased amine concentrations nor improved the oxidant-antioxidant balance in colonic digesta of piglets. However, both types of inulin efficiently reduced the activity of DNA repair enzymes.

## Introduction

Inulin-type fructans, including inulin and oligofructose, are plant-derived, water-soluble fructose polymers that result from sucrose metabolism and serve as storage carbohydrates. Native inulin derived from a fresh chicory (*Cichorium intybus* L.) root is a mixture of linear oligomers and polymers composed of fructose units linked by β(2 → 1) glycosidic bonds. The fructose chain is usually terminated with a glucose molecule linked through an α(1 → 2) bond, as in sucrose [1]. The length of the fructose chain ranges from 2 to 60 units and an average degree of polymerisation (DP) is 10–12 [2]. The short-chain fraction, with DP lower than 10, is called oligofructose and constitutes about 10% of native inulin [3]. Fractionation allows the removal of oligofructose and the production of long-chain inulin, the so-called ‘high performance’ inulin, with a chain length ranging from 11 to 60 and an average DP of around 25 [2].

The average daily consumption of inulin in humans ranges from 3 to 11 g in Europe and from 1 to 4 g in the USA. Wheat, onion, banana, garlic and leek are the most important sources of this polysaccharide in the diet [4]. Inulin in pig nutrition has been applied in dietary levels ranging from 1 to 8% [5–8]. This polysaccharide is fermented by intestinal microbiota to short-chain fatty acids [3] and was thought to act primarily as prebiotic improving host’s health by stimulating the growth of beneficial bacteria in the intestine [4]. Recently, it has been shown that inulin is an antioxidant exerting desirable effects in the liver of piglets at the level of 1–3% [9,10], *in vitro* [11] and *ex vivo* in human colonic mucosa exposed to lipopolysaccharide [12]. This activity of inulin may be of crucial importance for piglets during peri-weaning period, which is associated with health problems, such as intestinal bacteria overgrowth and diarrhoea [13], but also with oxidative stress [14–16]. Colonic epithelium is particularly susceptible to oxidative stress due to the prolonged passage time of digesta containing oxidised food particles, toxins and minerals participating in redox reactions [17]. Additionally, intestinal microbiota continuously generates reactive metabolites, such as amines playing a role in the synthesis of potentially carcinogenic *N*-nitrosocompounds [18] and contributing to oxidative stress caused by hydrogen peroxide accumulation during catabolism by polyamine oxidase [19]. Oxidative stress leads to the formation of different types of DNA adducts. These are 8-oxo-deoxyguanosine (8-oxodG) DNA adducts [17] and exocyclic etheno-DNA adducts, e.g. 1,*N*^6^-etheno-2’-deoxyadenosine (εdA) and 3,*N*^4^-etheno-2’-deoxycytidine (εdC), formed as a result of the reaction between lipid peroxidation products with DNA [20]. There are several DNA repair enzymes responsible for their removal, including oxo-guanine glycosylase, specific for 8-oxodG repair in eukaryotes [17], alkylpurine DNA *N*-glycosylase, specific for εdA, and thymine DNA glycosylase, which excises εdC adducts [21]. As an antioxidant, inulin may decrease the formation of DNA adducts in colonic tissue and affect the activity of DNA repair enzymes, but this effect has not been studied so far.

Previous studies demonstrated that DP and dietary level of inulin determined fructan content in the large intestine of piglets and that long-chain inulin had a more pronounced effect on microbial activity than short-chain inulin [8,22]. Moreover, *in vitro* studies revealed that scavenging activity of inulin toward free radicals also depended on its DP, but contrary to the effect on intestinal fermentation, it was greater for short-chain fructans [11]. Therefore, it was hypothesized that DP and dietary level of inulin could also determine its antioxidant properties *in vivo* and affect colonic environment and epithelium in piglets. The aim of the study was to verify the effect of the level of two inulin types in the diet on microbial formation of biogenic amines, oxidant-antioxidant balance in the digesta and DNA glycosylase activity in the colon of piglets offered experimental diets from pre- to post-weaning period. This nutritional approach was adopted to allow piglets become accustomed to solid feed, reduce weaning stress related to replacing sow milk with a cereal-based diet and enhance inulin effects.

## Materials and methods

### Ethics statement

The study complied with the principles of the European Union and the Polish Animal Protection Act and was approved by the 3^rd^ Local Animal Experimentation Ethics Committee (No. 30/2010, Warsaw University of Life Sciences-SGGW, Warsaw, Poland).

### Animals and experimental design

The experiment was performed on 56 castrated male PIC x Penarlan P76 piglets divided into 7 groups (n = 8), offered the following diets from the 10^th^ day of life: control–without inulin addition, supplemented with 1%, 2% or 3% native chicory inulin, with an average DP of 10 (IN10) or supplemented with 1%, 2% or 3% ‘high performance’ inulin, with an average DP of 23 (IN23) (Inulin Orafti GR or Orafti HPX, respectively; Beneo GmbH, Mannheim, Germany). All diets were cereal-based and inulin was introduced instead of corn starch (Table 1).

**Table 1:** Ingredient and nutrient composition of treatment diets.

Item	Control	IN10 diets^1^			IN23 diets^2^		
		1%	2%	3%	1%	2%	3%
**Composition (%)**							
Wheat	46.8	46.8	46.8	46.8	46.8	46.8	46.8
Barley	20	20	20	20	20	20	20
Corn starch	3	2	1	0	2	1	0
Inulin	0	1	2	3	1	2	3
Full fat soybean	5.9	5.9	5.9	5.9	5.9	5.9	5.9
Whey	9.7	9.7	9.7	9.7	9.7	9.7	9.7
Fish meal	4	4	4	4	4	4	4
Spray-dried plasma	4	4	4	4	4	4	4
Soybean oil	3.4	3.4	3.4	3.4	3.4	3.4	3.4
Calcium formate	0.3	0.3	0.3	0.3	0.3	0.3	0.3
Calcium carbonate	0.5	0.5	0.5	0.5	0.5	0.5	0.5
Calcium monophosphate	0.6	0.6	0.6	0.6	0.6	0.6	0.6
Sodium chloride	0.1	0.1	0.1	0.1	0.1	0.1	0.1
L-Lysine HCL (78,5%)	0.6	0.6	0.6	0.6	0.6	0.6	0.6
DL-Methionine (99%)	0.2	0.2	0.2	0.2	0.2	0.2	0.2
L-Threonine (98%)	0.3	0.3	0.3	0.3	0.3	0.3	0.3
L-Tryptophan (98%)	0.1	0.1	0.1	0.1	0.1	0.1	0.1
Mineral-vitamin mix^3^	0.4	0.4	0.4	0.4	0.4	0.4	0.4
Aroma	0.1	0.1	0.1	0.1	0.1	0.1	0.1
**Nutrients (% dry matter)**							
Dry matter	90.03	90.15	90.14	90.13	90.17	90.14	90.16
Crude ash	4.54	4.54	4.54	4.53	4.54	4.54	4.53
Crude protein	20.05	20.04	20.04	20.03	20.02	20.03	20.02
Ether extract	6.04	6.04	6.03	6.03	6.04	6.02	6.02
Crude fibre	1.52	1.52	1.51	1.51	1.52	1.52	1.51
Fructan	1.00	1.52	2.21	3.11	2.04	2.52	3.13
EM^4^, MJ/kg	14.3	14.3	14.3	14.3	14.3	14.3	14.3

^1^IN10 –inulin with an average degree of polymerisation of 10.

^2^IN23 –inulin with an average degree of polymerisation of 23.

^3^Supplied per kg of diet: vitamin A 2400 IU, vitamin D3 240 IU, vitamin E 12 mg, vitamin K3 480 μg, vitamin B1 480 μg, vitamin B2 960 μg, vitamin B6 960 μg, nicotinic acid 6.4 mg, pantothenic acid 3.2 mg, folic acid 640 μg, biotin 40 μg, vitamin B12 6.4 μg, choline chloride 48 mg, Mg 3.2 mg, Fe 24 mg, Zn 22.4 mg, Mn 9.6 mg, Cu 25.6 mg, I 0.16 mg, Se 64 μg, Co 64 μg

^4^EM–metabolisable energy. Concentrations (MJ/kg) in the diets were calculated based on ingredient composition and nutrient content.

In the pre-weaning period, the piglets were kept with their sows in farrowing pens on the farm (4 litters per group) and experimental diets were offered to them as a mash placed in a feeder. Feed intake was not measured in this period. At the 28^th^ day of life the piglets were weaned and weighed. Based on body weight two barrows from each litter were selected, ear-tagged and transported to the experimental facility. The average body weight of selected piglets was similar in each group (7.9 ± 1.4 kg). Upon arrival, the animals were divided according to dietary treatment and placed in pens, 4 animals each (2 pens per group). The piglets were kept under controlled conditions of 25°C and 12 h dark-light cycle, with free access to feed and water. Feed intake was measured once per week. After a 40 day-experimental period, the piglets were weighed, stunned by electric shock and exsanguinated at the 50^th^ day of life. The gastrointestinal tract was removed to collect digesta and tissue samples from the proximal, middle and distal colon.

### Chemical composition of diets

Nutrient contents in experimental diets were analysed according to standard procedures [23]. Fructan concentration was determined using the Fructan Assay Kit (Megazyme International Ireland, Bray, Ireland), according to the manufacturer’s protocol.

### Biogenic amine analysis

The digesta sample (2 g) was mixed thoroughly with 4 ml of water (18.2 MΩ) by vigorous vortexing and initially centrifuged at 1500xg for 15 min at 4°C. The obtained supernatant was collected and stored at -80°C until further analysis. After thawing it was cleared by centrifugation at 12,000xg for 10 min at room temperature and analysed for amine concentration using high-performance liquid chromatography and heptylamine as an internal standard, according to the previously described method [24]. Amines were derivatised with dansyl chloride and extracted using Waters SEP-PAK serif™ C18 cartridges for solid phase extraction (6 ml, 500 mg; Waters, Watford, Hertfordshire, UK). The separation was carried out using a Finnigan Surveyor Plus HPLC (Thermo Scientific, San Jose, USA) with a photodiode array detector set at 254 nm and a Waters Symmetry Shield RP_18_ column (150 × 3.9 mm i.d., 5 μm) preceded by a guard column (Waters Symmetry Shield RP_18_, 20 × 3.9 mm, 5 μm). Amine concentrations were calculated using standard curves prepared for individual compounds.

### Oxidant-antioxidant balance measurement

One gram of colonic digesta was sampled and stored at -20°C until analysis. The sample was thawed and homogenised in 0.8 ml of phosphate-citrate buffer (51.2 mM Na_2_HPO_4_, 49.7 mM citric acid, pH 5.0) for 30 s at high speed and centrifuged (10,000xg, 10 min, room temperature). The oxidant-antioxidant balance was then measured in a 96-well microplate according to the colorimetric method of Koliakos and Alamdari [25] based on simultaneous redox and enzymatic reactions, using 3,3’,5,5’-tetramethylbenzidine (TMB) and its cation. In this assay, the TMB cation was reduced by antioxidants and decolourized, while intact TMB was oxidized by peroxides to a colour cation [25]. The quantity of the TMB cation was measured spectrophotometrically and represented the oxidant-antioxidant balance in the digesta sample. The absorbance was measured at 450 nm, with a reference wavelength of 620 nm, using a Maxmat PL multidiagnostic platform (Erba Diagnostics France SARL, Montpellier, France). The balance values were calculated from a standard logarithmic curve prepared using 1 mM H_2_O_2_ as representative of the oxidants, and 59.5 mM uric acid as representative of the antioxidants. The standard solutions were mixed in varying proportions, from 100% 59.5 mM uric acid (standard 1) to 40% uric acid and 60% 1 mM H_2_O_2_ (standard 7). The results were expressed in arbitrary Hamidi-Koliakos (HK) units per gram of digesta. The HK unit is the percentage of H_2_O_2_ in the standard solution multiplied by 6. Thus, greater HK values indicate higher oxidant concentration in a sample.

### Nicking assay

Colon tissue samples were collected immediately after slaughter, frozen in liquid nitrogen and stored at -80°C until analyses of DNA repair enzyme activity. The tissues were homogenised in 4 volumes of 50 mM Tris-HCl buffer, pH 7.5, containing 1 mM EDTA and protease inhibitor cocktail. The cells were disrupted by sonication (3 x 15 s pulses with 30 s intervals), homogenate was centrifuged (7000xg, 4°C, 15 min), and the supernatant was collected and stored in aliquots at -80°C for further analyses.

Protein concentration was measured by the Bradford method using the Coomassie protein assay reagent (Sigma-Aldrich, Saint Louis, MO, USA). The activity of DNA glycosylases specific for 8-oxodG, εdA and εdC was measured by the nicking assay using a 5’-radiolabeled synthetic oligodeoxynucleotide duplex (Bioneer, South Korea) containing a single DNA adduct (8-oxodG, εdA or εdC), according to the previously described method [26]. In this assay, DNA adducts were excised from the duplex by DNA glycosylases present in the tissue extract and DNA was incised at the site of the removed base by apurinic/apyrimidinic endonuclease. The cleavage products of enzymatic reactions were separated in 20% polyacrylamide, 7 M urea denaturing gels and quantified using a Storm PhosphorImager and Molecular Dynamics ImageQuant 5.2 software (GE Healthcare, Waukesha, WI, USA). Enzyme activities were calculated from the linear part of the curve, reflecting the excision rate as a function of the amount of protein extract.

### Histological examination

Tissue samples from the colon were rinsed with 0.9% NaCl, fixed in Bouin’s solution and embedded in paraffin blocks. The samples were then sliced into 5-μm sections and stained with haematoxylin and eosin. Crypt depth and myenteron thickness were measured using a Zeiss Axio Star Plus (Carl Zeiss, Göttingen, Germany) light microscope and Axio Vision LE Rel. 4.5 (Carl Zeiss, 2002–2005) image analysis software. At least 30 measurements of each parameter were performed per one sample and individual means for each piglet were calculated.

### Statistical analyses

The data were analysed using one-way ANOVA and polynomial regression performed separately for control and IN10 groups, and for control and IN23 groups. Two-way ANOVA was performed to determine the effect of inulin type, inulin level and interaction, excluding the control group. The significance of differences between treatments were analysed using *post hoc* Tukey’s Honestly Significant Differences test. All analyses were performed using Statgraphics Centurion XVI ver. 16.1.03 (StatPoint Technologies, Inc., Warrenton, Virginia, USA) and the level of significance was set at P ≤ 0.05.

## Results

The pigs were healthy throughout the experiment and no signs of diarrhoea were observed. There was no effect of IN10 and IN23 levels on final body weight and body weight gain (Table 2). Feed intake was not subjected to statistical analysis, as it was measured only per pen.

**Table 2:** Feed intake and growth parameters of piglets. Data are presented as means and pooled SEM, and were calculated for the period from 28^th^ (weaning) to 50^th^ day of life.

Item	Control	IN10^1^			IN23^2^			SEM	P values^3^	
		1%	2%	3%	1%	2%	3%		IN10 level	IN23 level
Average daily feed intake, kg	0.53	0.54	0.59	0.48	0.46	0.60	0.72	0.03		
Final body weight, kg	18.2	17.5	20.1	18.6	18.4	18.7	17.8	0.37	0.352	0.946
Body weight gain, kg	9.9	9.4	10.4	10.0	9.1	10.0	8.9	0.24	0.714	0.630

^1^IN10 –inulin with an average degree of polymerisation of 10.

^2^IN23 –inulin with an average degree of polymerisation of 23.

^3^One-way ANOVA was performed separately for the control and IN10 groups and for the control and IN23 groups. Feed intake was not analysed statistically because it was estimated on a per-pen basis.

Methylamine, tryptamine, phenylethylamine, putrescine, cadaverine, 1,7-diaminoheptane, tyramine and spermidine were detected in each analysed colon segment (Table 3). Feeding the 2% and 3% IN10 diets increased tryptamine concentration (linear effect, P < 0.05) in the proximal colon of piglets in comparison with the control group. There was also a quadratic response of phenylethylamine concentration to IN10 level in a diet (P ≤ 0.05). In the middle colon, there was no effect of IN10 level on amine concentrations. In the distal colon, only methylamine was affected and its concentration was higher in piglets fed the 1% and 3% IN10 diets than in the control group (P < 0.05).

**Table 3:** Concentrations of biogenic amines (nM/g digesta) in colonic digesta of piglets fed diets differing in inulin types and levels. Data are presented as means and pooled SEM.

Amine	Control	IN10^1^			IN23^2^			SEM	P values^3^	
		1%	2%	3%	1%	2%	3%		IN10 level	IN23 level
**Proximal colon**										
Methylamine	69^a^	70	85	90	85^ab^	101^b^	72^ab^	3.5	0.303	0.031 Q
Tryptamine^4^	28^A^	39^AB^	50^B^	50^B^	47	44	29	2.3	0.042 L	0.068 Q
Phenylethylamine	19^a^	27	30	29	39^b^	35^ab^	24^ab^	1.6	0.094 Q	0.009 Q
Putrescine	207	489	397	332	276	489	385	48.7	0.522	0.298
Cadaverine	361	771	794	855	1062	888	598	77.2	0.185	0.103
1,7- Diaminoheptane^5^	34^a^	48	65	52	79^c^	59^bc^	45^ab^	3.4	0.181	0.000 Q
Tyramine	29	42	46	38	56	37	36	3.0	0.499	0.117
Spermidine	21	27	26	28	24	22	28	1.3	0.520	0.364
**Middle colon**										
Methylamine	58	70	76	62	74	73	70	4.1	0.626	0.746
Tryptamine	34	44	43	46	81	45	25	4.9	0.684	0.055
Phenylethylamine	29	35	34	37	38	33	31	1.7	0.589	0.551
Putrescine	122	460	220	87	202	141	299	47.6	0.326	0.281
Cadaverine	510	750	658	209	1058	447	740	103.7	0.399	0.448
1,7- Diaminoheptane^6^	60^a^	77	81	88	134^b^	117^ab^	71^ab^	6.7	0.574	0.025 Q
Tyramine	42	35	44	34	57	36	38	4.0	0.884	0.651
Spermidine^7^	25	27	25	32	26	20	30	1.2	0.153	0.252
**Distal colon**										
Methylamine	45^A^	75^B^	56^AB^	77^B^	67	75	69	3.5	0.041	0.053 Q
Tryptamine	29	35	45	31	38	51	44	2.7	0.295	0.233
Phenylethylamine	30	43	44	46	45	43	54	3.6	0.243	0.499
Putrescine	97	291	85	74	102	75	207	35.1	0.475	0.258
Cadaverine	673	470	409	147	1140	226	499	96.1	0.380	0.174
1,7-Diaminoheptane	86^a^	114	131	131	148^ab^	167^b^	99^ab^	7.2	0.264	0.008 Q
Tyramine	42	34	31	27	47	26	27	3.9	0.836	0.550
Spermidine	30	26	30	32	29	22	31	1.2	0.597	0.078

Means for the control and IN10 groups with different capital letters (A, B) differ significantly within rows at P < 0.05. Means for the control and IN23 groups with different lowercase letters (a, b) differ significantly within rows at P < 0.05.

^1^IN10 –inulin with an average degree of polymerisation of 10.

^2^IN23 –inulin with an average degree of polymerisation of 23.

^3^One-way ANOVA was performed separately for the control and IN10 groups and for the control and IN23 groups. L–linear effect at P ≤ 0.05; Q–quadratic effect at P ≤ 0.05.

^4^Effect of interaction between inulin type and level, P = 0.040.

^5^Effect of interaction between inulin type and level, P = 0.034.

^6^Effect of inulin type, P = 0.043; effect of interaction, P = 0.047.

^7^Effect of inulin level, P = 0.032.

In the proximal colon, piglets fed a diet with 1% and 2% of IN23 had higher phenylethylamine (P < 0.01) and methylamine (P < 0.05) concentrations, respectively, than the control group. Both IN23 diets also increased 1,7-diaminoheptane content (P < 0.001). The effect of IN23 level was quadratic for methylamine, tryptamine, phenylethylamine and 1,7-diaminoheptane (P ≤ 0.05). The concentration of 1,7-diaminoheptane was also higher in the middle (P < 0.05) and distal colon (P < 0.001) of piglets fed the 1% and 2% IN23 diet, respectively. In both segments the effect of IN23 level on this amine was quadratic (P ≤ 0.05), as well as for methylamine in the distal colon (P ≤ 0.05). Other amines were not affected by dietary supplementation with IN23.

Interaction between DP and level of inulin (P < 0.05) affected tryptamine and 1,7-diaminoheptane concentrations in the proximal colon (P < 0.05). Feeding the 2% and 3% IN10 diets increased tryptamine in comparison with the 1% IN10 diet, while feeding the 2% and 3% IN23 diets reduced the tryptamine content as compared to the 1% IN23 diet. The concentration of 1,7-diaminoheptane gradually decreased with increasing IN23 level, whereas the effect of IN10 level on this amine was irregular. In the middle colon 1,7-diaminoheptane content was greater in piglets fed IN23 than IN10 diets and there was an interactive effect (P < 0.05). In animals fed IN10 diets 1,7-diaminoheptane concentration was directly proportional to inulin level, while in piglets fed IN23 diets the relationship was opposite. Inclusion level affected spermidine concentration which was greater in piglets fed diets with 3% than 2% inulin (P < 0.05). In the distal colon there were no effects of DP, dietary level or interaction on amine concentrations.

Dietary supplementation with IN10 and IN23 did not change the oxidant-antioxidant balance in colonic digesta of piglets (Table 4). The balance value was higher only in the distal part of the colon in animals fed the 1% IN10 diet compared to those offered the 3% IN10 diet (P ≤ 0.05) and there was a quadratic response to increasing IN10 level in a diet (P ≤ 0.05). There were no effects of DP, dietary level or interaction on the oxidant-antioxidant balance in any part of the colon.

**Table 4:** Oxidant-antioxidant balance (arbitrary HK units/g digesta) in colonic digesta of piglets fed diets differing in inulin types and levels. Data are presented as means and pooled SEM.

Segment	Control	IN10^1^			IN23^2^			SEM	P values^3^	
		1%	2%	3%	1%	2%	3%		IN10 level	IN23 level
Proximal colon	133	175	175	167	191	150	204	10.7	0.479	0.373
Middle colon	157	156	158	157	172	233	179	9.3	1.000	0.132
Distal colon	147^AB^	182^B^	154^AB^	134^A^	160	254	161	10.3	0.050 Q	0.065

Means for the control and IN10 groups with different capital letters (A, B) differ significantly within row at P ≤ 0.05.

^1^IN10 –inulin with an average degree of polymerisation of 10.

^2^IN23 –inulin with an average degree of polymerisation of 23.

^3^One-way ANOVA was performed separately for the control and IN10 groups and for the control and IN23 groups. Q–quadratic effect at P ≤ 0.05.

**Fig 1:**
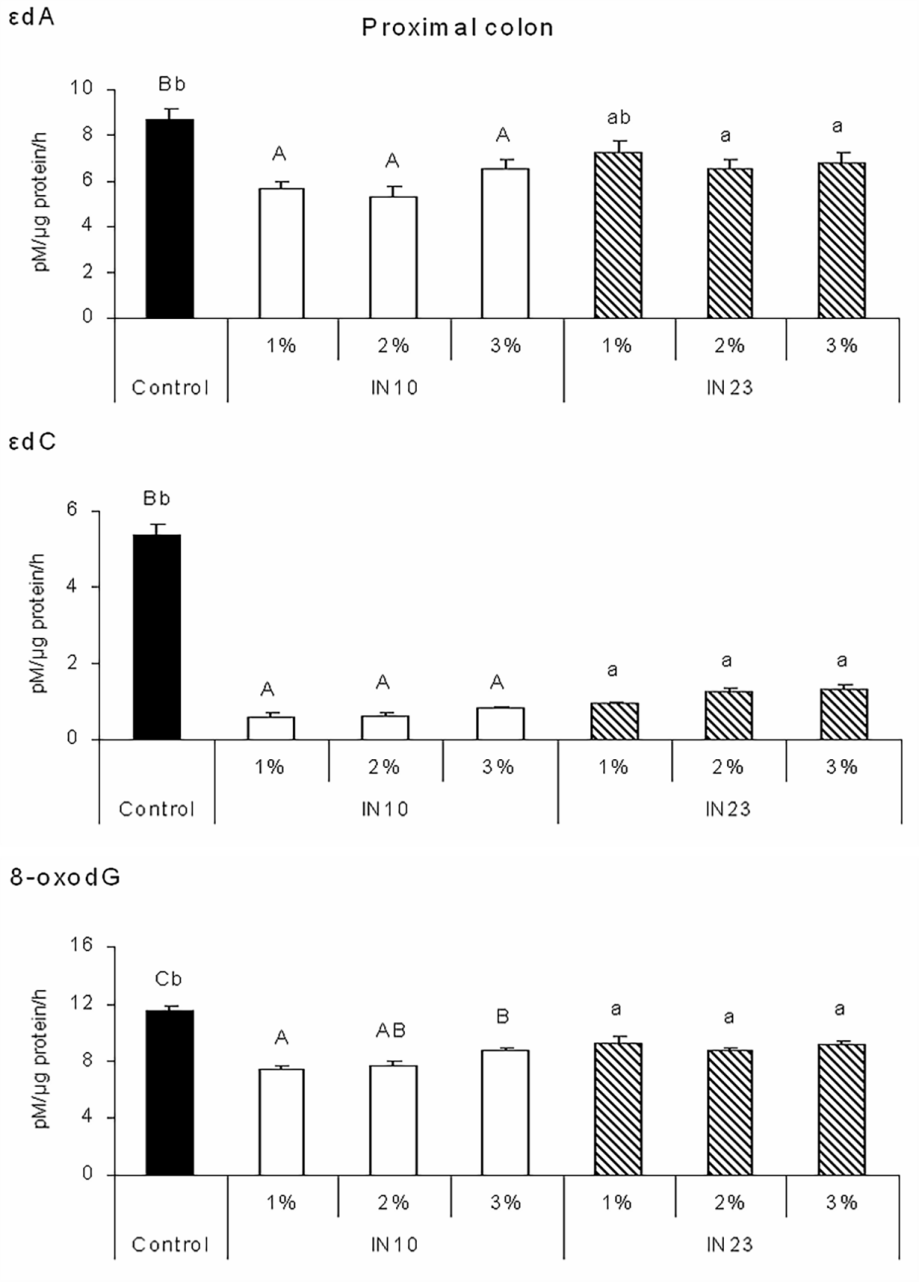
Effects of IN10 and IN23 levels on the activity of DNA glycosylases involved in the excision of εdA, εdC and 8-oxodG DNA adducts in the proximal colon of piglets. Data are presented as means ± SEM. One-way ANOVA was performed separately for control and IN10 groups and for control and IN23 groups. Means for control and IN10 groups with different capital letters (A, B, C) differ significantly at P < 0.001. Means for control and IN23 groups with different lowercase letters (a, b) differ significantly at P ≤ 0.001 (P < 0.05 for εdA). All responses to IN10 and IN23 levels were quadratic at P < 0.0001 (the IN23 effect for εdA was quadratic at P < 0.01). Two-way ANOVA was performed excluding control group. Inulin type affected: εdA (P = 0.005), εdC (P < 0.001) and 8-oxodG (P < 0.001). Inulin level affected εdC (P = 0.011) and 8-oxodG (P = 0.037), and interaction affected 8-oxodG (P = 0.034). IN10, native chicory inulin with an average degree of polymerisation of 10; IN23, ‘high performance’ inulin with an average degree of polymerisation of 23; εdA, 1,*N*^6^-etheno-2’-deoxyadenosine; εdC, 3,*N*^4^-etheno-2’-deoxycytidine; 8-oxodG, 8-oxo-deoxyguanosine.

**Fig 2:**
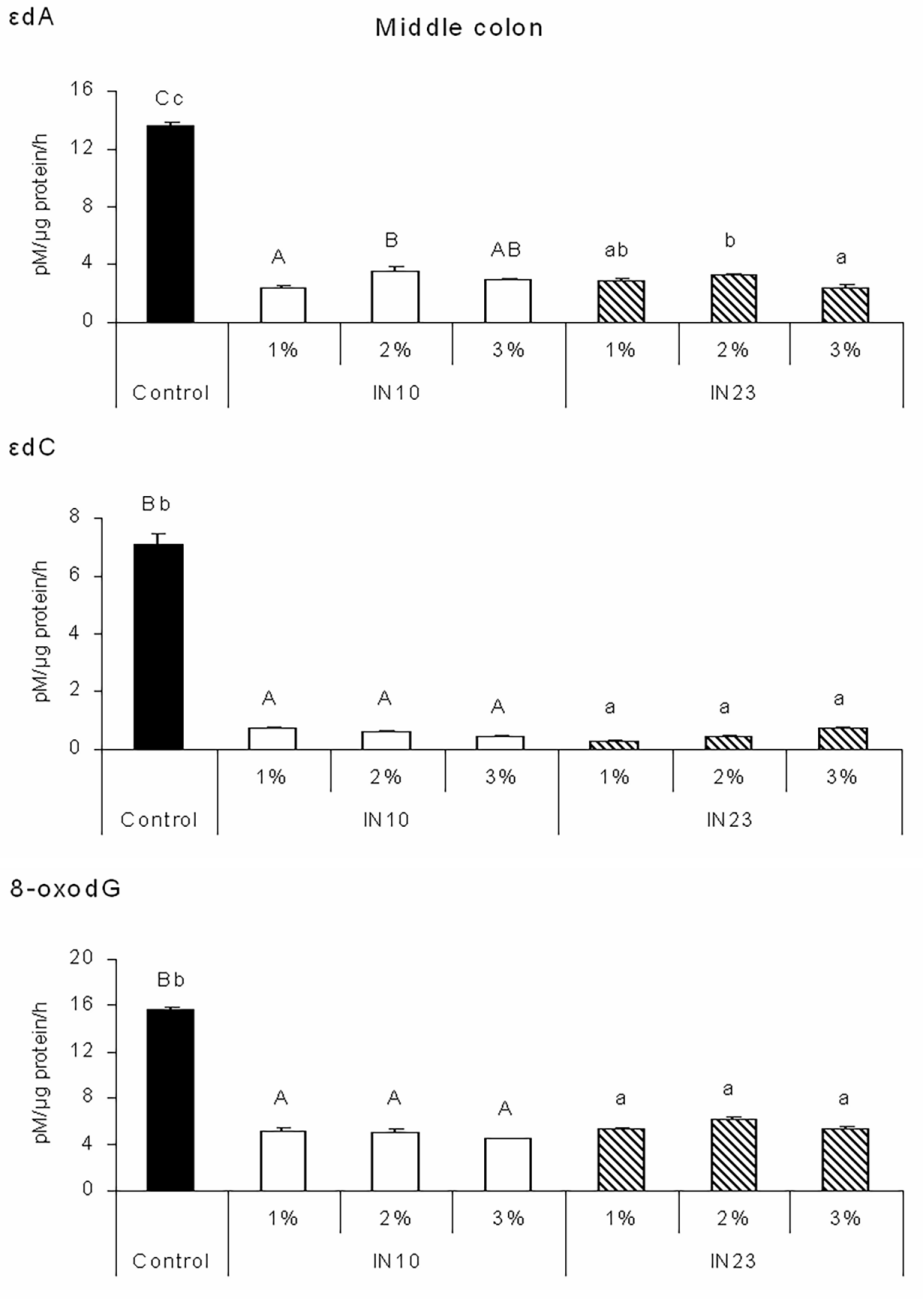
Effects of IN10 and IN23 levels on the activity of DNA glycosylases involved in the excision of εdA, εdC and 8-oxodG DNA adducts in the middle colon of piglets. Data are presented as means ± SEM. One-way ANOVA was performed separately for control and IN10 groups and for control and IN23 groups. Means for control and IN10 groups with different capital letters (A, B, C) differ significantly at P < 0.001. Means for control and IN23 groups with different lowercase letters (a, b) differ significantly at P ≤ 0.001. All responses to IN10 and IN23 levels were quadratic at P < 0.001. Two-way ANOVA was performed excluding control group. Inulin type affected: εdC (P = 0.018) and 8-oxodG (P = 0.001). Inulin level affected εdA (P < 0.001) and 8-oxodG (P = 0.020), and interaction affected εdA (P = 0.037) and εdC (P < 0.001). IN10, native chicory inulin with an average degree of polymerisation of 10; IN23, ‘high performance’ inulin with an average degree of polymerisation of 23; εdA, 1,*N*^6^-etheno-2’-deoxyadenosine; εdC, 3,*N*^4^-etheno-2’-deoxycytidine; 8-oxodG, 8-oxo-deoxyguanosine.

**Fig 3:**
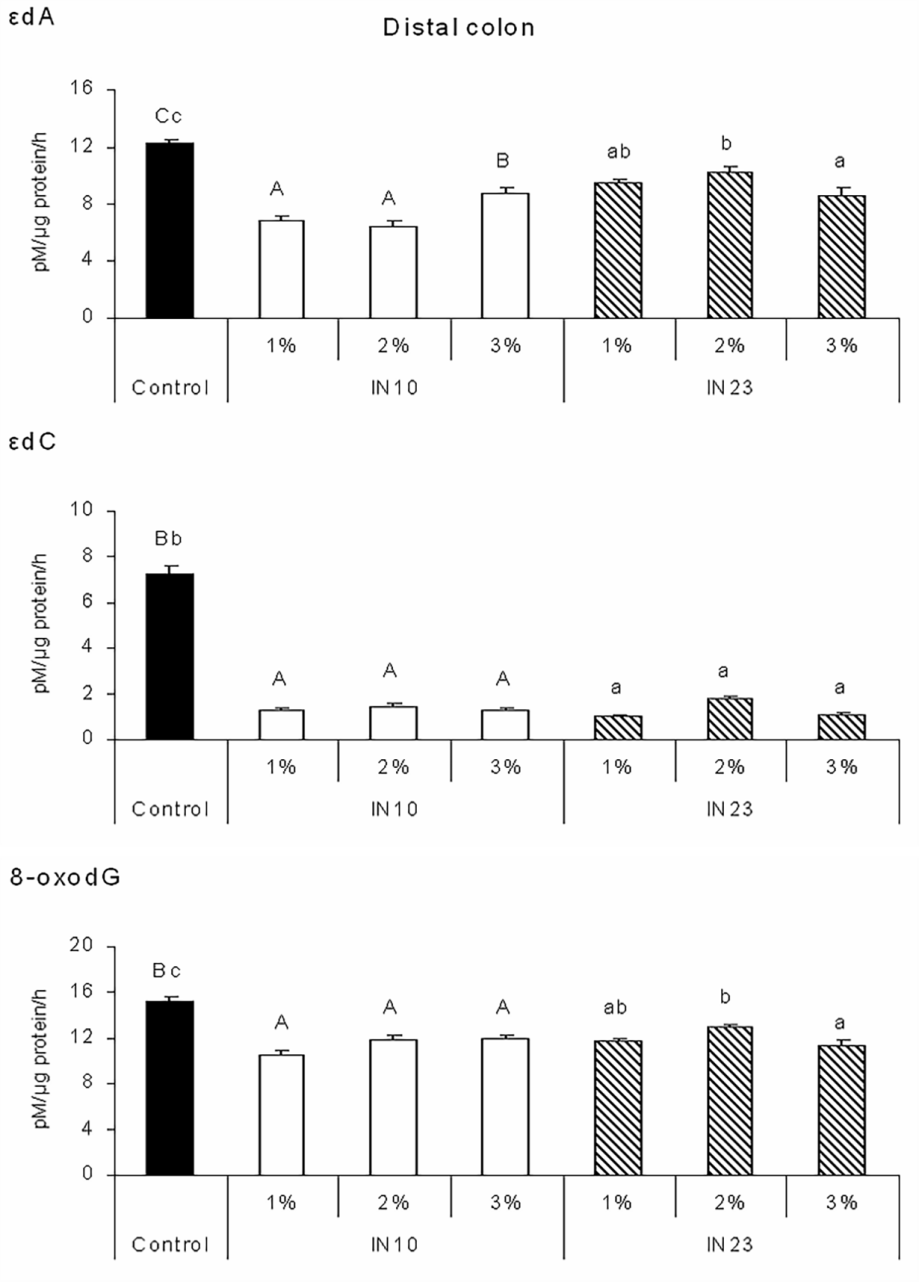
Effects of IN10 and IN23 levels on the activity of DNA glycosylases involved in the excision of εdA, εdC and 8-oxodG DNA adducts in the distal colon of piglets. Data are presented as means ± SEM. One-way ANOVA was performed separately for control and IN10 groups and for control and IN23 groups. Means for control and IN10 groups with different capital letters (A, B, C) differ significantly at P < 0.001. Means for control and IN23 groups with different lowercase letters (a, b) differ significantly at P ≤ 0.001. All responses to IN10 and IN23 levels were quadratic at P < 0.0001. Two-way ANOVA was performed excluding control group. Inulin type affected εdA (P < 0.001), inulin level affected εdC (P < 0.001) and 8-oxodG (P = 0.006), and interaction affected εdA (P < 0.001) and εdC (P = 0.014). IN10, native chicory inulin with an average degree of polymerisation of 10; IN23, ‘high performance’ inulin with an average degree of polymerisation of 23; εdA, 1,*N*^6^-etheno-2’-deoxyadenosine; εdC, 3,*N*^4^-etheno-2’-deoxycytidine; 8-oxodG, 8-oxo-deoxyguanosine.

The activities of DNA glycosylases involved in the excision of oxidative DNA adducts, i.e. 8-oxodG, εdA and εdC in different parts of piglet colon are shown in Figs 1, 2 and 3. Piglets fed IN10 diets had lower εdA excision activity in the proximal, middle and distal colon as compared with the control group (P < 0.001). Excision activity specific for εdA was also affected by IN23 level. In the proximal colon, it was reduced by the 2% and 3% IN23 diets (P < 0.05), while in the middle and distal colon by all IN23 diets in comparison with control (P < 0.001). Feeding with all IN10 and IN23 diets reduced εdC and 8-oxodG excision activities in each part of the colon (P < 0.001). All effects of IN10 and IN23 levels were quadratic (P < 0.01).

Moreover, IN10 diets reduced activities of DNA repair enzymes in the proximal colon (Fig 1) in comparison with IN23 diets (P < 0.001). Piglets fed diets with 3% inulin had higher εdC and 8-oxodG excision activity than those fed diets with 1% and 2% inulin, respectively (P < 0.05). In the proximal colon, interaction between inulin DP and level affected only 8-oxodG excision activity (P < 0.05). It was lower in piglets fed the 2% and 3% IN23 diets than in those fed the 1% IN23 diet, while in animals offered the IN10 diets the 2% and 3% addition of inulin increased the activity as compared to diet supplemented with 1% inulin. In the middle colon (Fig 2), excision activity specific for εdC was higher in piglets fed IN10 than IN23 diets (P < 0.05), but the effect of DP on 8-oxodG excision activity was opposite (P < 0.05). Feeding diets with 2% inulin increased activity of DNA glycosylase specific for εdA as compared to other inulin levels (P < 0.001), and for 8-oxodG as compared to the 3% level (P < 0.05). There was an interactive effect on εdA and εdC excision activities (P < 0.05 and P < 0.001, respectively). In piglets fed IN10 diets there was an inversely proportional relationship between εdC excision activity and inulin level, while in those fed IN23 diets the relationship was directly proportional. As for the activity of DNA glycosylase specific for εdA, it was lower in piglets fed the 2% and 3% IN23 diets but higher in those fed the 1% IN23 diet as compared to the respective IN10 diets.

In the distal part of the colon (Fig 3), diets supplemented with 2% inulin increased εdC excision activity in comparison to other diets (P < 0.001), while IN10 diets reduced εdA excision activity as compared to IN23 diets (P < 0.001). Activities of DNA glycosylases specific for these adducts were also affected by the interaction. In general, piglets fed IN23 diets had lower εdC excision activity than those offered IN10 diets, but it was not a true for animals receiving diets supplemented with 2% inulin (P < 0.05). In contrast, feeding the 1% and 2% IN23 diets to piglets increased εdA excision activity in comparison to the respective IN10 diets, but there was no difference between animals fed diets supplemented with 3% inulin (P < 0.001).

Histological parameters of the proximal, middle and distal colon of piglets are shown in Table 5. There were no effects of feeding IN10 or IN23 diets on crypt depth or myenteron thickness in the colon. Two-way ANOVA also did not show any effects of experimental factors on histological measurements.

**Table 5:** Histological parameters (μm) in colon segments of piglets fed inulin-supplemented diets. Data are presented as means and pooled SEM.

Parameter	Control	IN10^1^			IN23^2^			SEM	P values^3^	
		1%	2%	3%	1%	2%	3%		IN10 level	IN23 level
**Proximal colon**										
Crypt depth	403	391	420	402	417	413	405	5.7	0.525	0.924
Myenteron thickness	411	397	398	364	463	428	408	12.7	0.770	0.710
**Middle colon**										
Crypt depth	475	470	475	471	479	457	476	9.8	0.998	0.955
Myenteron thickness	439	455	442	396	450	413	444	12.0	0.650	0.861
**Distal colon**										
Crypt depth	539	565	541	545	538	541	556	11.1	0.909	0.979
Myenteron thickness	430	395	389	377	378	437	395	9.2	0.511	0.134

^1^IN10 –inulin with an average degree of polymerisation of 10.

^2^IN23 –inulin with an average degree of polymerisation of 23.

^3^One-way ANOVA was performed separately for the control and IN10 groups and for the control and IN23 groups.

## Discussion

Colonic epithelium can be damaged by endogenous factors produced within the cells, compounds circulating in the blood or digesta constituents [27]. Many of them can cause oxidative stress generated when reactive oxygen species production exceeds the capacity of the antioxidant defence system. Under basal conditions, antioxidant enzymes are fully functional and sufficient to maintain oxidative DNA damage, but not reduce it in the event of increased production of reactive oxygen species. It makes the colonic environment prooxidant and predisposes cells to damage [17]. The colonic environment may be improved through the reduction of proteolytic fermentation leading to the formation of toxic compounds such as ammonia, phenolic and indolic compounds, hydrogen sulphide or biogenic amines [18]. Attempts have been made to modulate the colonic environment through dietary interventions involving different indigestible carbohydrates [28–30], but none of them have been fully successful in reducing biogenic amine production. In the present study, the incorporation of inulin into pig diets also did not reduce amine concentration in the colon, regardless of the fructose chain length. What is more, feeding a diet supplemented with inulin may even increase concentrations of some amines, as shown for tryptamine, methylamine, phenylethylamine and 1,7-diaminoheptane. The effect of inulin depended on dietary level, chain length and colon segment and was consistent with previous findings [8], which demonstrated that IN23 increased the concentration of branched-chain fatty acids, i.e. markers of proteolytic fermentation [18]. The results are also in line with other studies indicating variable effects of fermentable carbohydrates on amine concentration. Inclusion of 0.9% oligofructose in the adult dog diet did not affect amine production [29], while 12% sugar beet pulp in the weaned piglet diet reduced the total amine concentration in the proximal colon, but not in the distal colon [30]. Incorporation of 7% resistant starch [28] in growing pig diets increased phenylethylamine, putrescine and tyramine concentrations in the large intestine compared to pectin and cellulose. Varying effects of fermentable carbohydrates on amine concentration may be related to their availability as energy substrates for microbiota, which can be a source of fermentable protein themselves [31]. Thus, the increased microorganism mass, caused by feeding diets containing inulin, could have contributed to higher concentrations of this putrefactive compounds observed in the present study.

The current study showed no effect of inulin level on the concentrations of putrescine and spermidine, which are polyamines synthesised by prokaryotes during arginine and ornithine catabolism [32]. Desquamated colonocytes are other source of these compounds in the intestinal lumen [18], but in the present study there was no effect of inulin level on crypt depth, which indicated that epithelial cell renewal was unaffected and could explain the results of polyamine analysis.

To the best of our knowledge, this is the first study, in which an attempt was made to measure the oxidant-antioxidant balance in colonic digesta using the colorimetric method of Koliakos and Alamdari [25]. The results confirmed the prooxidant environment in the colon and showed that inulin, regardless of the chain length, did not improve it. The results of *in vitro* studies showed that inulin types with lower DP and higher water solubility had greater antioxidant capability than inulin with higher DP and lower solubility [11]. Despite higher fructan content in digesta of piglets fed IN10 diets, as previously described [8], the balance values were not reduced by this inulin type. Disparities may be ascribed to different analytical approaches, since TMB and its cation were used in the Koliakos-Alamdari method, whereas in *in vitro* studies of Pasqualetti et al. [11] antioxidative capability of inulin was tested using 2,2’-azinobis-3-ethylbenzothiazoline-6-sulphonic acid monocationic radical. Therefore, it is possible that applying the latter substrate in the assay of the oxidant-antioxidant balance could reveal beneficial effect of inulin. On the other hand, incorporating inulin in a diet might exert beneficial systemic effects, despite the lack of desirable changes in the large intestinal environment and microbial activity [8,9,33,34]. As for the colon, recent findings have demonstrated that inulin alleviated negative effects of oxidative stress induced by lipopolysaccharide [11,12]. In the current study, no challenge was applied, which could increase the production of reactive oxygen species in the colon tissue of piglets above the basal level. Nonetheless, feeding all inulin-supplemented diets considerably reduced the activity of DNA glycosylases, which are key enzymes in the process of base excision repair [35]. These unique results might indicate that inulin directly reduced oxidative stress in colonocytes, induced by the prooxidant environment in the colon, and in consequence, the formation of DNA adducts excised by DNA glycosylases. Both IN10 and IN23 reduced not only 8-oxodG excision activity, but also that of εdA and εdC, which are etheno-DNA adducts formed in the reaction of DNA with lipid peroxidation products [20]. These results are consistent with former findings indicating decreased concentration of thiobarbituric acid-reactive substances, a biomarker of lipid peroxidation, in the liver of piglets fed inulin-supplemented diets [9]. The results obtained so far have indicated that, in the case of oxidative stress induced in the colon, inulin restored the level of DNA-(apurinic or apyrimidinic site) lyase [12], which is the DNA base excision repair protein and redox signalling factor maintaining transcription factors in an active reduced state [36]. This finding suggests that decreased excision activities in piglets resulted from the reduced level of DNA adducts and not from the decreased expression of genes encoding DNA repair enzymes. However, confirmation of the beneficial effect of inulin on the colon would require analysis of gene expression to exclude its down-regulation, which may lead to excessive accumulation of DNA damage in cells [37].

In summary, feeding inulin-supplemented diets neither decreased the concentrations of biogenic amines nor improved the oxidant-antioxidant balance in colonic digesta of piglets, which could make the large intestinal environment less prooxidant. Nevertheless, both types of inulin reduced the activity of DNA glycosylases involved in the excision of 8-oxodG, εdA and εdC, indicating the antioxidant effect of these dietary supplements and reduced formation of DNA adducts in colon tissue.

## Supporting information

S1 Table Body weight of piglets and amine concentrations, oxidants-antioxidants balance, DNA glycosylase activity and histological parameters of the colon. Raw data.
(XLS)
